# Right Unilateral Versus Bilateral Electroconvulsive Therapy in Patients With Clinical Depression

**DOI:** 10.7759/cureus.18313

**Published:** 2021-09-27

**Authors:** Maham Saeed, Zainab Sher, Faryal Khan, Fizza Iqbal, Taha Ahmad Siddiqui, Abdul Wahab, Izza Khalid, Khizer Shamim, Jawed Akbar Dars, Anoosh Farooqui, Kiran Abbas

**Affiliations:** 1 Department of Medicine, Jinnah Postgraduate Medical Centre, Karachi, PAK; 2 Department of Psychiatry, Civil Hospital Karachi, Karachi, PAK; 3 Department of Internal Medicine, Jinnah Sindh Medical University, Karachi, PAK; 4 Department of Medicine, Dow Medical College, Karachi, PAK; 5 Department of Medicine, Jinnah Sindh Medical University, Karachi, PAK; 6 Department of Medicine, Abbasi Shaheed Hospital, Karachi, PAK; 7 Department of Medicine, Fatima Jinnah Medical University, Lahore, PAK; 8 Department of Medicine, Ziauddin University, Karachi, PAK; 9 Department of Psychiatry, Jinnah Postgraduate Medical Centre, Karachi, PAK; 10 Department of Surgery, Jinnah Sindh Medical University, Karachi, PAK; 11 Department of Surgery, United Medical and Dental College, Karachi, PAK

**Keywords:** depression, seizure, right unilateral, bilateral, electroconvulsive therapy

## Abstract

Introduction

Electroconvulsive therapy (ECT) is a functional treatment for a significant mental illness that involves a momentary application of electrical stimulation to induce generalized seizures. The use of right unilateral (RUL) and bilateral (BL) ECT has been controversial. Thus, the study aimed at comparing the effectiveness of RUL ECT and BL ECT in treating depression.

Methodology

A longitudinal study was conducted between September 2016 and January 2021 at a tertiary care hospital in Sindh, Pakistan. All patients over the age of 18 with clinically diagnosed depression in the last month were included in the study. Baseline depression scores and post-treatment scores were determined using Hamilton Depression Rating Scale (HDRS). All patients were assigned to each treatment group. Group A was administered right unilateral electroconvulsive therapy, while group B was administered bilateral electroconvulsive therapy. Adverse effects were documented right after treatment, at four hours, and then one day after therapy. Depression severity was determined after each ECT session using the HDRS scale. Electroconvulsive therapy was discontinued when an HDRS score of 10 was achieved.

Results

The mean HDRS score at baseline in the bilateral ECT group was 24.99 ± 3.938, which lowered to 17.56 ± 2.65 by the 3rd session, 12.45 ± 3.76 by the 6th session, and to 11.86 ± 2.3 by the end of treatment (p<0.0001). Similarly, the right unilateral ECT was equally effective in improving the depressive symptoms (p<0.0001). There was no significant difference between the efficacy of bilateral and unilateral placements of electrodes in electroconvulsive therapy (p=0.116).

## Introduction

Electroconvulsive therapy (ECT) is a functional treatment for a significant mental illness that involves a momentary application of electrical stimulation to induce generalized seizures [1]. ECT had been commonly practiced to treat a range of psychiatric illnesses, peculiarly severe depression; however, it has been replaced by oral medications in recent years [2]. 

A recently published review by Pinna et al. revealed that ECT is a profound therapeutic strategy in patients with severe and refractory neuropsychiatric illnesses [3]. 

Despite the fact that ECT has been in practice for over 45 years, there is still debate over the mental illnesses for which it is appropriate, its effectiveness in treating them, the appropriate means of administration, potential consequences, and the amount to which it is used in different contexts [4] Reservations regarding the possibility for ECT overuse and abuse, as well as a need to protect patients' rights, have arisen as a result of these difficulties. Simultaneously, there is apprehension that restricting ECT usage in reaction to public opinion and legislation may deny some patients of a presumably beneficial treatment [5].

In the recent past few decades, researchers have focused their efforts on determining the efficacy of ECT and its indications, as well as understanding its molecular mechanisms, determining the degree of side effects, and determining the best treatment approach [6,7]. 

Unfortunately, in Pakistan, only a few studies have been conducted exploring the efficacy of ECT in patients with severe psychiatric ailments [8]. Minhas et al. revealed that out of the 5240 patients, 1520 (29%) were administered ECT as a therapeutic modality. Of these, 60.8% had depression. The rate of discontinuation against medical advice was 16.7% [8]. The reasons for discontinuation were severe headaches, muscle soreness, intolerable nausea or vomiting, and indifference in the symptoms. There is a dire need for further exploration in different techniques of administering ECT and determining the most optimum and safe method as current literature has been inconsistent. Keeping this in mind, the current study was conducted in a tertiary care center where modified ECT was offered to patients with psychiatric ailments. The authors compared the effectiveness of right unilateral (RUL) ECT against bilateral (BL) ECT in treating depression.

## Materials and methods

A longitudinal study was conducted between September 2016 and January 2021 at a tertiary care hospital in Sindh, Pakistan. Ethical approval was obtained prior to the initiation of the study (reference # JSMU/IRB/2019/-456). Informed consent from all patients was taken. If a patient was not oriented at presentation, the consent was taken from their family. All patients diagnosed with depression between the age of 18 and 65 years were included in the study. 

Patients with multiple comorbidities, neurological disease, and a history of psychotic disorders were excluded from the research. All patients had their absolute, and relative contraindications to ECT evaluated [9]. Demographic data, baseline investigations, and information about medication were collected with the help of proforma. 

ECTs were administered by senior postgraduates who had more than two years of experience in the department. All patients received multiple ECT sessions overall and a minimum of two sessions per week. 

Before ECT, the procedure was narrated comprehensively in the local languages to all patients by the evaluators. The ECT device was prepared. Patients in group A, i.e., bilateral ECT, had electrodes placed at the bitemporal region. While patients in group B, i.e., right unilateral ECT, had electrodes placed on the right temporal region. Propofol was used as the anesthetic agent before administration of the ECT (modified ECT). 

Every adverse effect was documented immediately, 12 hours, and 24 hours after therapy. At baseline, the Hamilton Depression Rating Scale (HDRS) was used to gauge progress in depressive symptoms among the individuals after the third, sixth session of ECT, and one month post-intervention [10]. 

Those who discontinued treatment against medical advice were excluded from the study. The main reason for the discontinuation of treatment was indifference in the symptoms. By the end of the study, only 404 out of 481 were left who completed all sessions of ECT. A discontinuation rate of 18.4% was documented. 

Version 26 of the Statistical Package for Social Sciences (IBM Inc., Armonk, New York) was used to analyze the data. Frequencies and percentages were calculated to determine categorical variables like gender and side effects. For continuous factors such as age and the number of ECTs delivered, a mean with standard deviation was computed, as well as HDRS scores at each follow-up. To determine the efficacy of each study group, before and after intervention paired t-tests were applied at a 5% level of significance. The significant cut-off was established at a p-value of less than 0.05.

## Results

A sum of 404 respondents' data was examined. About 15%, i.e., 61/404 patients, had depression with psychotic features. The mean age of patients in the bilateral electroconvulsive therapy (ECT) group was 34.62 ± 10.64 years, while in the unilateral ECT group, the mean age was 33.96 ± 8.76 years. In the bilateral ECT group, 85 (42.08%) were males, and 117 (57.92%) were females. In the unilateral ECT group, 88 (43.9%) were male, and 114 (56.10%) were females. 

The mean Hamilton Depression Rating Scale (HDRS) score at baseline in the bilateral ECT group was 24.99 ± 3.938, which lowered to 17.56 ± 2.65 by the third session, 12.45 ± 3.76 by the sixth session, and to 11.86 ± 2.3 by the end of treatment (p<0.0001). Similarly, the right unilateral ECT was equally effective in improving the depressive symptoms (p<0.0001) (Table 1).

**Table 1:** Improvement in mean HDRS with respect to electroconvulsive therapy in patients with depression ECT - electroconvulsive therapy; HDRS - Hamilton Depression Rating Scale

	HDRS score (mean ± S.D)	P-value
Bilateral ECT group		
Baseline	24.99 ± 3.938	<0.0001
After the third session	17.56 ± 2.65	
Baseline	24.99 ± 3.93	<0.0001
After the sixth session	12.45 ± 3.76	
Baseline	24.99 ± 3.93	<0.0001
Post-intervention	11.86 ± 2.3	
Unilateral ECT group		
Baseline	25.73 ± 4.865	<0.0001
After the third session	16.64 ± 978	
Baseline	25.73 ± 4.865	<0.0001
After the sixth session	12.54 ± 3.56	
Baseline	25.73 ± 4.865	<0.0001
Post-intervention	11.34 ± 2.45	

There was no significant difference between the efficacy of bilateral and unilateral placements of electrodes in ECT (p=0.116) (Table 2).

**Table 2:** Mean depression scores in bilateral ECT group versus unilateral ECT group ECT - electroconvulsive therapy; HDRS - Hamilton Depression Rating Scale

HDRS scores	Bilateral ECT group	Unilateral ECT group	P-value
Pre-intervention			
Mean ± SD	24.99 ± 3.938	25.73 ± 4.865	0.534
Post-intervention			
Mean ± SD	11.86 ± 2.3	11.34 ± 2.45	0.116

## Discussion

The study looked at the effectiveness of right unilateral and bilateral electroconvulsive treatment in patients with depression. In the treatment of depression, we discovered that both BL and RUL ECT were highly effective. We were unable to detect any significant difference between the two techniques of electroconvulsive therapy. 

Van et al. conducted a study on patients who presented with major depression and had not yet achieved the required response after 12 RUL ECT sessions. The authors concluded that continuing with RUL ECT for six months after 12 sessions will give a better outcome. A favorable response was also achieved by combining antidepressants with RUL or BL ECT [11]. Similarly, Sackeim et al. found that the second dose of high dose, brief pulse, and bitemporal ECT had a higher response on depressed patients who were not responsive to the first dose of ECT [12]. The authors support the conclusion that patients might benefit from longer courses of electroconvulsive therapy.

In developed countries, modified electroconvulsive therapy has been used for a long time, but in developing countries, unmodified ECT remained in practice until it was prohibited in 2017 [13]. Previously modified ECT was commonly used in parts of India and in some African countries where anesthetic resources limited practice [14]. However, with time, unmodified ECT was replaced with modified ECT owing to the advancements in the field of anesthesia. According to Panwala et al.'s study, ECT is one of the most successful therapies for depression in Nigeria, and there is a need to raise knowledge about the issue among health practitioners [15]. In a study published by Cambridge University Press, ECT was reported to be highly beneficial against not only depression but also effectively treated schizophrenia and postpartum psychosis [16]. Memory impairment was a commonly reported side effect. 

The current study evaluated the optimum placement of ECT (bilateral versus right unilateral). There are comparable data in favoring bilateral ECT; however, the current study revealed that both techniques were extremely effective in alleviating depressive symptoms. In a study published in the Journal of Psychiatric Research, the authors evaluated the therapeutic effectiveness of right unilateral and bilateral electroconvulsive treatment. The efficacy did not significantly differ between the groups; however, individuals in the RUL group had significantly lower rates of confusion and blood pressure disturbances. Furthermore, patients in the RUL group performed significantly better with respect to cognition and auditory memory [17]. 

We faced certain limitations during the study. About 16 percent of patients in our study discontinued the treatment against medical advice and were lost to follow-up. The main reason for discontinuation was no improvement in symptoms of depression. Further studies should be focused on combination therapy, and factors affecting patient compliance should be explored.

## Conclusions

We observed that both RUL and BL ECT provided relief from depressive symptoms, effectively. The present study did not observe any significant differences in efficacy between the two electroconvulsive therapy techniques. Future studies should observe long-term follow-up to assess the recurrence rate of depression in both techniques. Overall, ECT is also effective besides being safe for treating depression on both a right unilateral and a bilateral basis.

## References

[1] Shah AJ, Wadoo O, Latoo J (2013). Electroconvulsive therapy (ECT): important parameters which influence its effectiveness. BJMP.

[2] McCall WV (2001). Electroconvulsive therapy in the era of modern psychopharmacology. Int J Neuropsychopharmacol.

[3] Pinna M, Manchia M, Oppo R (2018). Clinical and biological predictors of response to electroconvulsive therapy (ECT): a review. Neurosci Lett.

[4] Coffey CE, Weiner RD (1990). Electroconvulsive therapy: an update. Hosp Community Psychiatry.

[5] Mihaljević-Peleš A, Bajs Janović M, Stručić A, Šagud M, Skočić HanŽek M, Živković M, Janović Š (2018). Electroconvulsive therapy - general considerations and experience in Croatia. Psychiatr Danub.

[6] Payne NA, Prudic J (2009). Electroconvulsive therapy part I: a perspective on the evolution and current practice of ECT. J Psychiatr Pract.

[7] American Psychiatric Association (2008). The practice of electroconvulsive therapy: recommendations for treatment, training, and privileging (A task force report of the American Psychiatric Association). https://books.google.ee/books/about/The_Practice_of_Electroconvulsive_Therap.html?id=iuuLJtmo_EYC&source=kp_book_description&redir_esc=y.

[8] Minhas HM, Ostroff R (2012). Practice of electroconvulsive therapy in a tertiary care hospital in Pakistan. J ECT.

[9] Baghai TC, Möller HJ (2008). Electroconvulsive therapy and its different indications. Dialogues Clin Neurosci.

[10] Obeid S, Abi Elias Hallit C, Haddad C, Hany Z, Hallit S (2018). Validation of the Hamilton Depression Rating Scale (HDRS) and sociodemographic factors associated with Lebanese depressed patients. Encephale.

[11] van Duist M, Spaans HP, Verwijk E, Kok RM (2020). ECT non-remitters: prognosis and treatment after 12 unilateral electroconvulsive therapy sessions for major depression. J Affect Disord.

[12] Sackeim HA, Prudic J, Devanand DP (2020). The benefits and costs of changing treatment technique in electroconvulsive therapy due to insufficient improvement of a major depressive episode. Brain Stimul.

[13] Hanlon C, Tesfaye M, Wondimagegn D, Shibre T (2010). Ethical and professional challenges in mental health care in low- and middle-income countries. Int Rev Psychiatry.

[14] Gallegos J, Vaidya P, D'Agati D (2012). Decreasing adverse outcomes of unmodified electroconvulsive therapy: suggestions and possibilities. J ECT.

[15] Panwala ZZ, Dadarwala DD, Mehta RY (2020). The impact of a “brief ECT orientation module” on the knowledge and attitude of nursing students toward electroconvulsive therapy. Ann Indian Psychiatry.

[16] Shukla GD (1981). Electroconvulsive therapy in a rural teaching general hospital in India. Br J Psychiatry.

[17] Dominiak M, Antosik-Wójcińska AZ, Goetz Z, Sikorska O, Stefanowski B, Gorostiza D, Święcicki Ł (2021). Efficacy, safety and tolerability of formula-based unilateral vs bilateral electroconvulsive therapy in the treatment of major depression: a randomized open label controlled trial. J Psychiatr Res.

